# Improving myocardial infarction diagnosis with Siamese network-based ECG analysis

**DOI:** 10.1371/journal.pone.0313390

**Published:** 2025-01-30

**Authors:** Vaibhav Gadag, Simrat Singh, Anshul Harish Khatri, Shruti Mishra, Sandeep Kumar Satapathy, Sung-Bae Cho, Abishi Chowdhury, Amrit Pal, Sachi Nandan Mohanty

**Affiliations:** 1 School of Computer Science and Engineering, Vellore Institute of Technology, Chennai, Tamil Nadu, India; 2 Centre for Advanced Data Science, Vellore Institute of Technology, Chennai, Tamil Nadu, India; 3 Department of Computer Science, Yonsei University, Seoul, South Korea; 4 School of Computer Science & Engineering (SCOPE), VIT-AP University, Amaravati, Andhra Pradesh, India; University of Minnesota, UNITED STATES OF AMERICA

## Abstract

**Background:**

Heart muscle damage from myocardial infarction (MI) is brought on by insufficient blood flow. The leading cause of death for middle-aged and older people worldwide is myocardial infarction (MI), which is difficult to diagnose because it has no symptoms. Clinicians must evaluate electrocardiography (ECG) signals to diagnose MI, which is difficult and prone to observer bias. To be effective in actual practice, an automated, and computerized detection system for Myocardial Infarction using ECG images, must meet a number of criteria.

**Objective:**

In an actual clinical situation, these requirements—such as dependability, simplicity, and superior decision-making abilities—remain crucial. In the current work, we have developed a model using a dataset that consists of a combination of 928 ECG images taken from publicly available Mendeley Data. It was converted into three classes Myocardial Infarction, Abnormal heartbeat, and Normal.

**Methods:**

The dataset is then imported, pre-processed, and split into a 70:20:10 ratio of training, validation, and testing. It is then trained using the Siamese Network Model.

**Results:**

The classification accuracy comes out to be 98%. The algorithm works excellently with datasets having class imbalance by taking pair of images as input. The validation and testing classification matrix is then generated and the evaluation metrics for both of them come out to be a near-perfect value.

**Conclusion:**

In this study, we developed the ECG signals based early detection of cardiovascular diseases with Siamese network model.

## 1. Introduction

The most common cause of mortality worldwide is now cardiovascular diseases (CVDs), surpassing cancer. Myocardial infarction (MI), angina, and arrhythmia are examples of CVDs, which are conditions of the heart and blood vessels. With characteristics like sudden start and rapid changes that put patients’ lives in peril, this disease can be well classified as one of the most commonly occurring and acute CVDs today. Myocardial infarction (MI), sometimes known as a "heart attack," refers to the death or damage of heart tissue brought on by a lack of blood flow. The terms "myocardial" and "cardinal" relate to the heart and muscles, respectively, and the word "infarction" refers to tissue death. Patients are unable to prepare as a result, making the illness more dangerous and deadlier with a high death rate as a result [1]. Early detection and diagnosis are therefore essential to prevent the negative effects of MI, such as heart failure, arrhythmia, and death [2].

Any of the currently available techniques, such as an electrocardiogram (ECG), echocardiography, magnetic resonance imaging (MRI), and others, can be used to diagnose a MI patient. Since the ECG is a non-invasive, low-cost main equipment that is routinely used in hospitals to detect cardiac abnormalities, it is considered as the initial MI diagnostic tool for patients in critical circumstances. A diagnostic method called an electrocardiogram (ECG) employs electrodes placed on the chest to track the human heart’s electrophysiology throughout time. It helps in diagnosis of Myocardial Infraction (MI) since it can swiftly analyse the morphological alterations connected to any myocardial infarction. However, patients with MI may pass away due to a manual and erroneous identification of cardiac anomalies. To help the doctor identify MI from ECG signals quickly and accurately, automated detection with high recall performance metric is essential. With the robust pace of technological evolution, especially the advancements in deep learning-based computer-assisted methods, it is now possible to optimize 12-lead ECG data for MI and other cardiac ailments. The existence and location of MI are both shown by the ECG. Inversion of T-waves, ST-segment elevation, and distorted appearance of Q-waves are all signs of MI. These are frequently used to categorize feature vectors [3]. Furthermore, the ECG offers a rapid and extremely accurate diagnosis of heart failure when performed and read properly.

Models based on deep learning and machine learning techniques may be used to differentiate between MI and normal at the subject level. Machine learning’s discrete sequential feature extraction and classification procedures may call for intricate hand-crafted technical concerns. In comparison, DL automates and integrates feature extraction and categorization. The DL models is often made up of an Artificial Neural Network that can automatically pull-out significant characteristics from high-dimensional raw data using its hidden layers. Among its tens to hundreds of hidden layers, the deep convolutional neural network (DCNN), a form of DL model, contains several convolutional, pooling, and fully-connected layers. To extract features, the convolutional kernels convolve the input signals. A consistent resolution of feature map is maintained by the pooling layer, while lowering the computational cost of the network [4]. Two common types of pooling layers are max-pooling and average pooling. The classification process’s output is done by the fully linked last layer of the DCNN. Finding and classifying early illness course variations using DL models frequently yields excellent results [5].

In this study, we used one such network architecture named as Siamese Network Model (SNN). A Siamese neural network, also known as a twin neural network, is an artificial neural network that consists of two or more subnetworks that are similar to one another in terms of configuration, parameters, and weights. The other subnetworks often utilize the same setup even though only one of them has been trained. These networks compare the feature vectors of the inputs to determine how similar they are. A neural network has a property of predicting different classes. However, this presents a challenge, when we need to add or remove new classes from the data. The neural network must be updated in this situation and retrained using the entire dataset. Deep neural networks require a lot of data to train on as well. SNNs, however, are trained using a similarity function. So, we can teach it to determine whether the two images are identical. As a result, we can classify new types of data without having to retrain the network. We also faced challenges with class inequality and a dearth of photographs about this topic. But with the help of One-shot learning, Siamese Networks can learn to recognize a small number of photos from each class in the future. This made it more appropriate for our circumstances. Additionally, as its learning mechanism differs slightly from that of classification, averaging it with a classifier can achieve results that are far superior to those achieved by averaging two correlated supervised models, as was the case in many of the earlier works on the subject. Even though it takes more training time and requires parameter fine-tuning, we still achieved decent performance metrics given that we used raw ECG images from Mendeley Data. We had three classes: one for myocardial infarction, one for irregular heartbeats, and one for ECG pictures of a typical person’s heartbeat. To train the Siamese model, we divided the data into train, test, and validation groups. We then evaluated the model on various evaluation metrics.

The rest of the paper is organized as follows: a brief survey is done in Section 2, on earlier works on the diagnosis of MI in patients; in Section 3, the architecture of the model is explained; methodology and implementation, in Section 4; results in Section 5; discussion in Section 6; Ablation Study in Section 7; and conclusion, in Section 8.

The key points for consideration:

The early identification and classification of Myocardial Infarction using ECG graphs of patients is the main goal of paper. In doing so, we have taken a novel approach by using Siamese Neural Networks (SNN). To the best of our knowledge no research has yet employed SNN for the above task. Below are a few points which highlights the area our study covers:

✓ Firstly, the study will add to the existing literature on MI diagnosis using ECG. This will provide researchers with a better understanding of this somewhat elusive condition.
✓ Secondly, to show that in the real world where patients’ ECG data is minimal, the Siamese neural network can be used as it can be a better fit in classification tasks.
✓ Thirdly, to provide a detailed review of existing works using various techniques, and how our work differs from them by analysing the performance of a new algorithm when tested upon medical datasets.
✓ Finally, the study opens up a new paradigm to explore research opportunities in the fight against various Cardiovascular diseases. The limitations and future works can be taken up further by researchers in getting a breakthrough treatment, thus, helping humanity as a whole.

## 2. Literature survey

Some of the recent ECG classification research using machine learning methods has some produced excellent performance metrics. For the same reasons and aim, various sorts of these approaches have been studied and evaluated in by Rai *et al*. [6] suggested a unique detection system for Myocardial Infarction, by employing ECG signals as input to a hybrid CNN-Long short-term memory network (LSTM). In another study. by Jahmunah *et al*. [7], designed two models using DenseNet and CNN respectively in order to categorize patients among ten classifications of MI and healthy subjects depending on the region of cardiac involvement. They used pre-processed ECG signals from PTB database [8] were used to extract ECG beats using an R peak detection technique. The two models were then each were given an input of ECG signals. The outputs of both models were then subjected to the enhanced class activation mapping (CAM) to enhance the visualization made by the models of the precise ECG which in return improved the predicted judgments, for the required 11 classes. Rawi *et al*. [9] in their study very interestingly discussed the contemporary methods used in the ECG devices, and how Convolutional Neural Network have been examined and investigated in order to ensure better results in reading an ECG. In a study by Chakraborty *et al*. [10] conducted a thorough comparison of a few existing ideas using various ways to detect and predict myocardial infarction. Then, in order to attain greater performance, they presented a deep learning strategy for future deployment.

This study by Joloudari *et al*. [11] evaluated AI-based techniques for MI diagnosis based on ECG and other biological data. Effective Deep convolutional neural networks (DCNNs) produced great classification performance for MI diagnosis. In this paper, Baloglu *et al*. [12] discussed a DL model with a holistic structure on conventional 12-lead ECG data was proposed for MI detection. A convolutional neural network (CNN) is the most often utilised approach. The trained CNN model with the suggested architecture achieved extremely high accuracy and sensitivity for diagnosis. A study by Sraitih [3] investigated the performance of three supervised machine learning models, SVM, KNN, and random forest (RF), in distinguishing normal and MI cases using real ECG data from the publicly available PTB database but all the models performed similar and didn’t yield any positive conclusion. Whereas the research by Li *et al*. [13] offered to develop an SLC-GAN, which is an automated MI prediction model that uses Generative Adversarial Networks for the creation of single-lead ECG data with excellent morphological similarity. By utilizing CNNs with ECG, it was able to identify MI automatically and generated ECG signals from the GAN. Thus, as per the experimental results, conventional MI classification methodologies were outperformed by this proposed technique of SLC-GAN. With 5-fold cross-validation, SLC-MI GAN’s classification accuracy of over 90%. The study about Myocardial Infarction by Sahoo *et al*. [14] aimed to create a classification framework for electrocardiogram (ECG) signals that uses morphological, temporal domain, and empirical mode decomposition (EMD) features to differentiate between MI and healthy control (HC). The entire experiment was tested using the PTBDB database, the classifier obtains an accuracy of 98.75 per cent over feature fusion.

In more studies like [15] it was observed in a variety of CNN models, that it is possible to increase the accuracy of classification by combining support vector machines (SVM) and linear discriminant analysis (LDA). And rather than manual feature extraction in the study [16] it was concluded that how deep neural networks are capable of performing feature extraction right from the data. Furthermore, it has been shown that feature maps produced by a deep neural network trained on a large number of generic input images can be utilized as universal ECG signal spectrogram descriptors and produce features that allow arrhythmia classification. It was also discovered in [16] the training dataset can be enlarged, which can improve deep neural network performance. They conducted tests on sizable datasets collected under uncontrolled circumstances, demonstrating the precision and resilience of Deep-ECG under less-than-ideal circumstances which lead to positive results in their study.

These were the most recent research works done in the diagnosis of Myocardial Infarction (MI) [17] using ECG signals. In our study, the motivation for conducting research was to have an accurate and automated system to detect Myocardial Infarction (MI) from ECG signals, as it reduces observer bias and enhances early diagnosis. Experimental assumptions were the availability of labeled ECG datasets and the feasibility of training deep learning models [18] over a cloud-based GPU environment, namely Google Colab with NVIDIA T4. It was assumed that the ECG images used are representative of general, real-world clinical scenarios, ensuring both generalization and robustness of the model [19].

It was learned that [20] looks at models based on acoustic data for detecting COVID-19 using cough and speech sounds, addressing issues like data variability and privacy concerns. Whereas [21] examines EEG-based emotion recognition, focusing on computational intelligence methods and the need for personalized models to account for differences among subjects. And [22] reviews advancements in deep learning for image classification, noting improvements in model architectures like CNNs, and challenges related to computational efficiency and interpretability. Overall, these studies emphasize the need for diverse and high-quality datasets, effective feature engineering, balancing model complexity with interpretability, and the potential for personalized, real-time applications.

## 3. Architecture of proposed model

**Fig 1:**
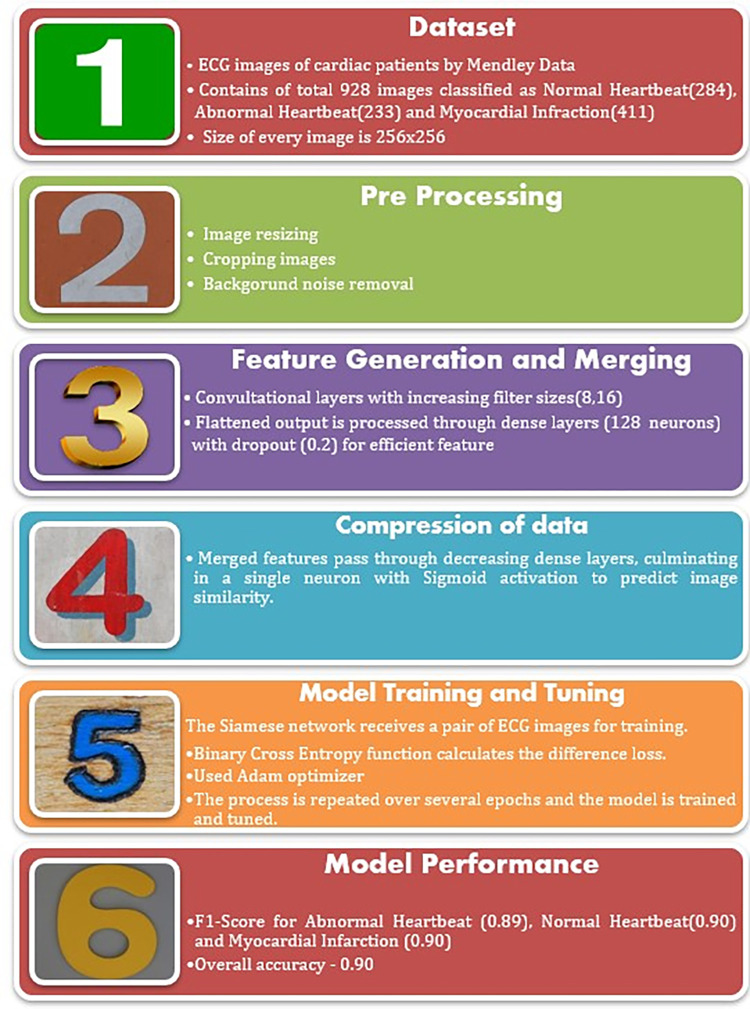
Detailed proposed model.

**Fig 2:**
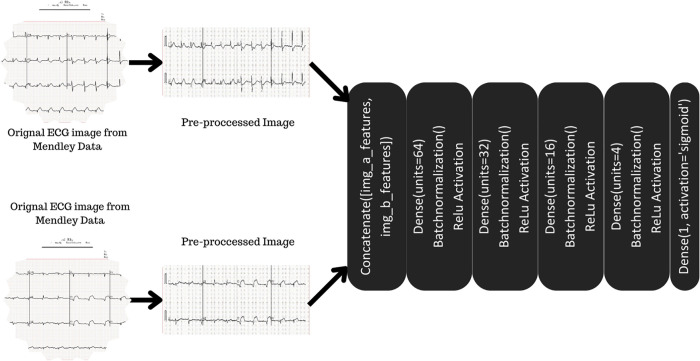
Overall proposed model.

The Figs 1 and 2 represents the Model used for the analysis of the ECG i.e Siamese network. Instead of the learning to classify the images, Siamese network learns to differentiate between two input images and also the similarity between both images. The model consists of two neural networks which are identical to each other. Both of them are given the same image as input. The output of both networks is passed through the flattened layer and then to the other layers such as the dense and activation layers. Binary cross-entropy was the loss function, optimizer was Adam and activation functions used was Relu and Linear function.

## 4. Mathematical model

The Siamese Network Model is designed with two identical convolutional neural networks (CNNs) that process pairs of ECG images at the same time. Each network extracts features from the images using convolutional layers with ReLU activation and max-pooling layers to reduce the size of the data. These features are then flattened and passed through dense layers, ultimately generating a similarity score using a sigmoid activation function.

The loss function employed is binary cross-entropy, which is calculated as shown in Eq (1):

$$L(y,\hat{y})=-\frac{1}{N}\sum_{i=1}^{N}\left[y_{i}\;\log\left(\hat{y_{i}}\right)+(1-y_{i})log(1-\hat{y_{i})}\right]$$ (1)

y:actuallabel(0,1)

$$\hat{y}:\;predicted\;similarity\;score$$

N:numberoftrainingsamples

The networks share weights to ensure consistent feature extraction. The Adam optimizer is used to minimize the loss function and update the model parameters. This architecture effectively captures similarities between pairs of ECG signals, enhancing the detection of Myocardial Infarction (MI) by learning from both similar and dissimilar image pairs.

### a) Implementation

**Fig 3:**
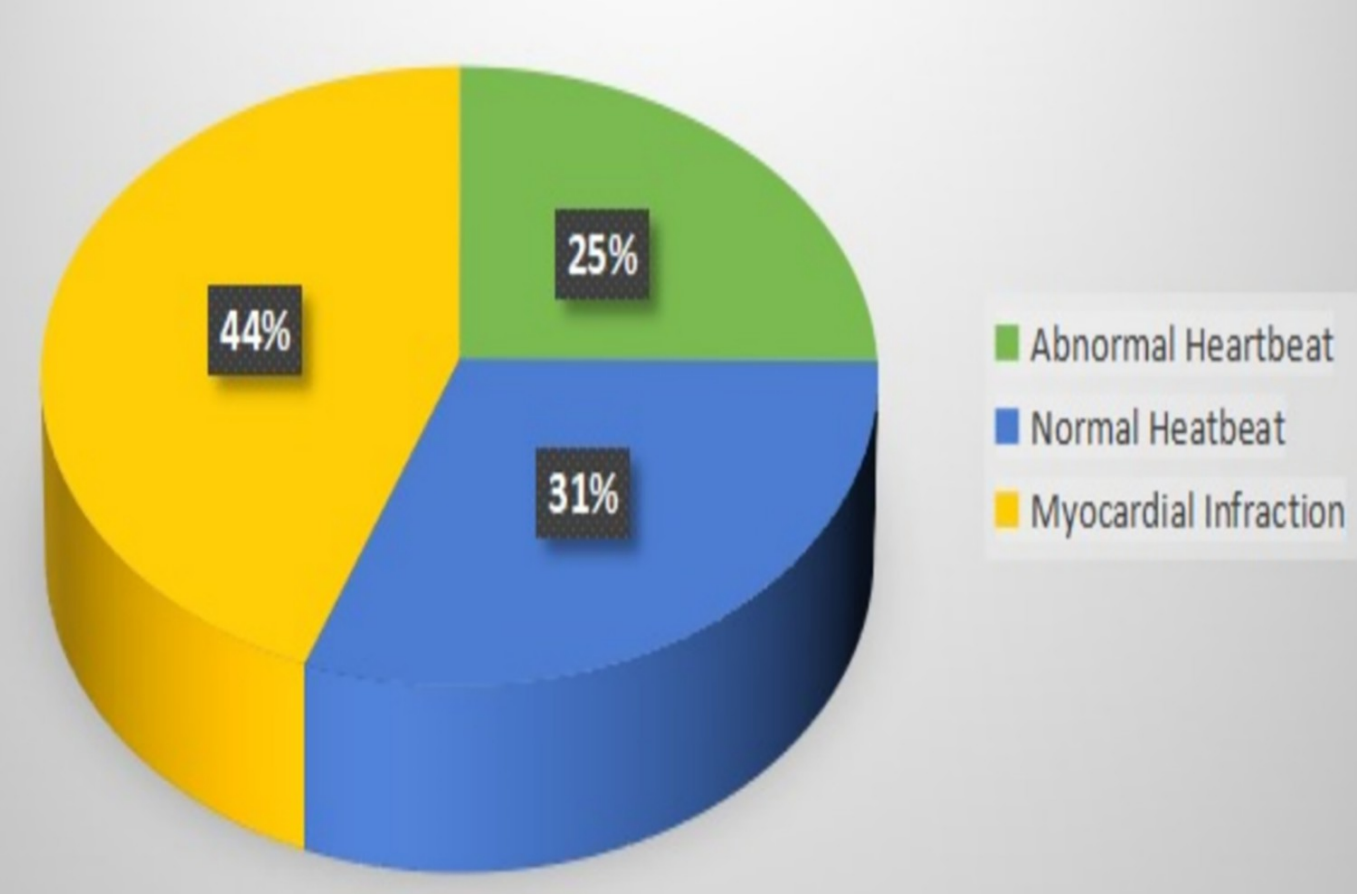
Distribution of dataset.

**Fig 4:**
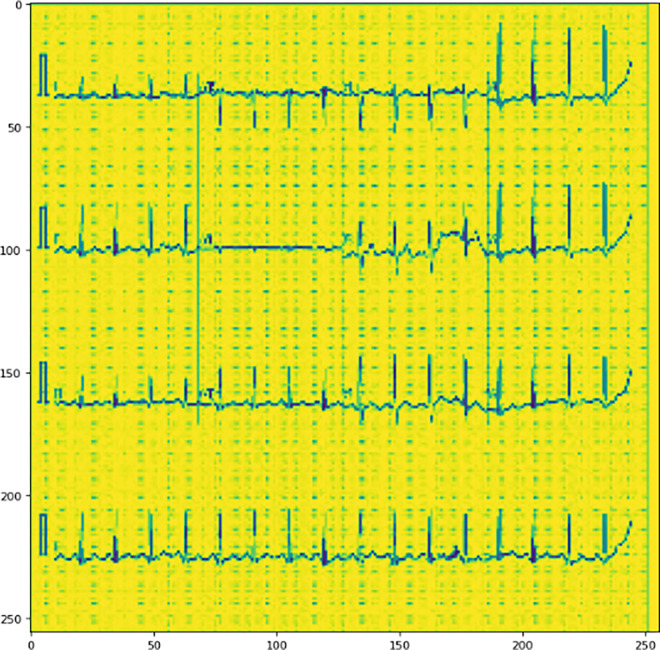
Represents the sample image of the ECG which is used for the purpose of training.

**Fig 5:**
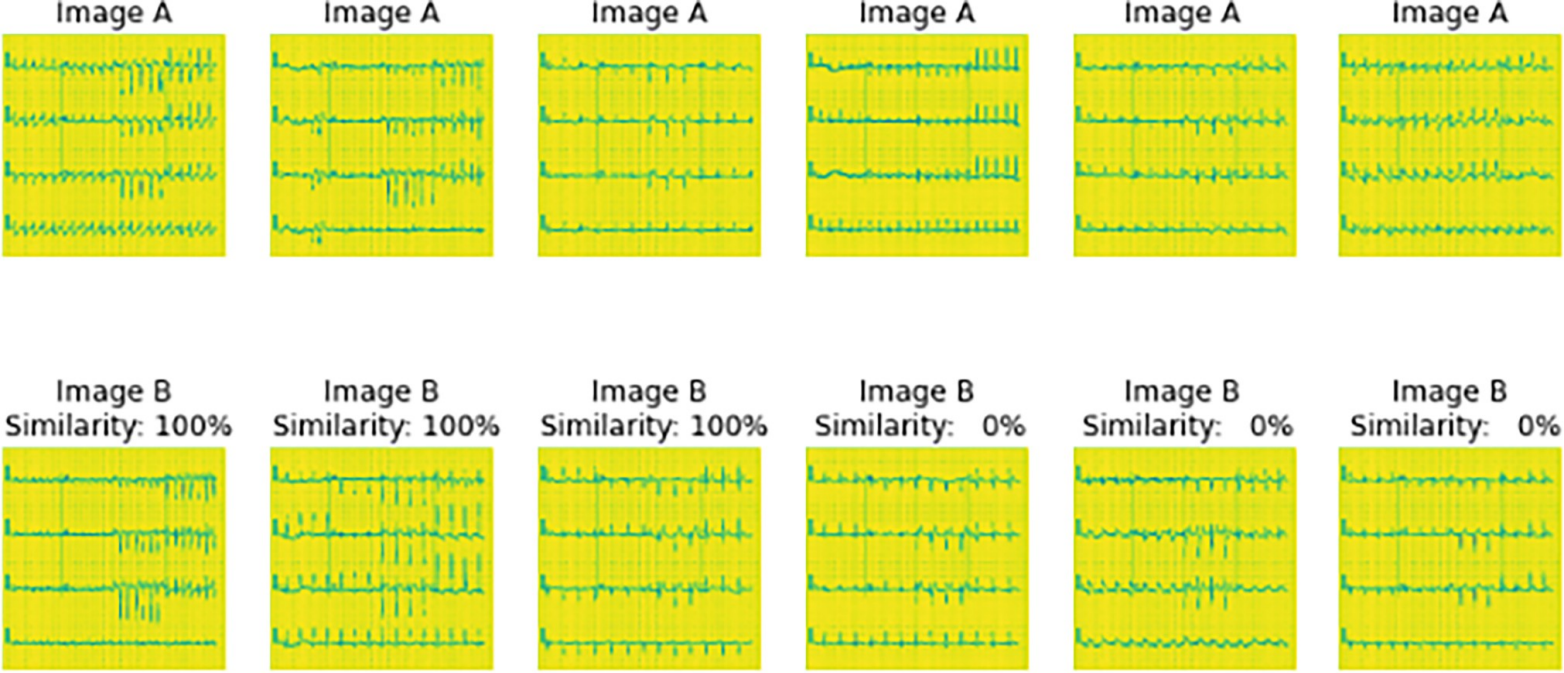
Represents the images that will feed to the untrained Siamese model i.e generating sample images to feed the untrained model.

**Fig 6:**
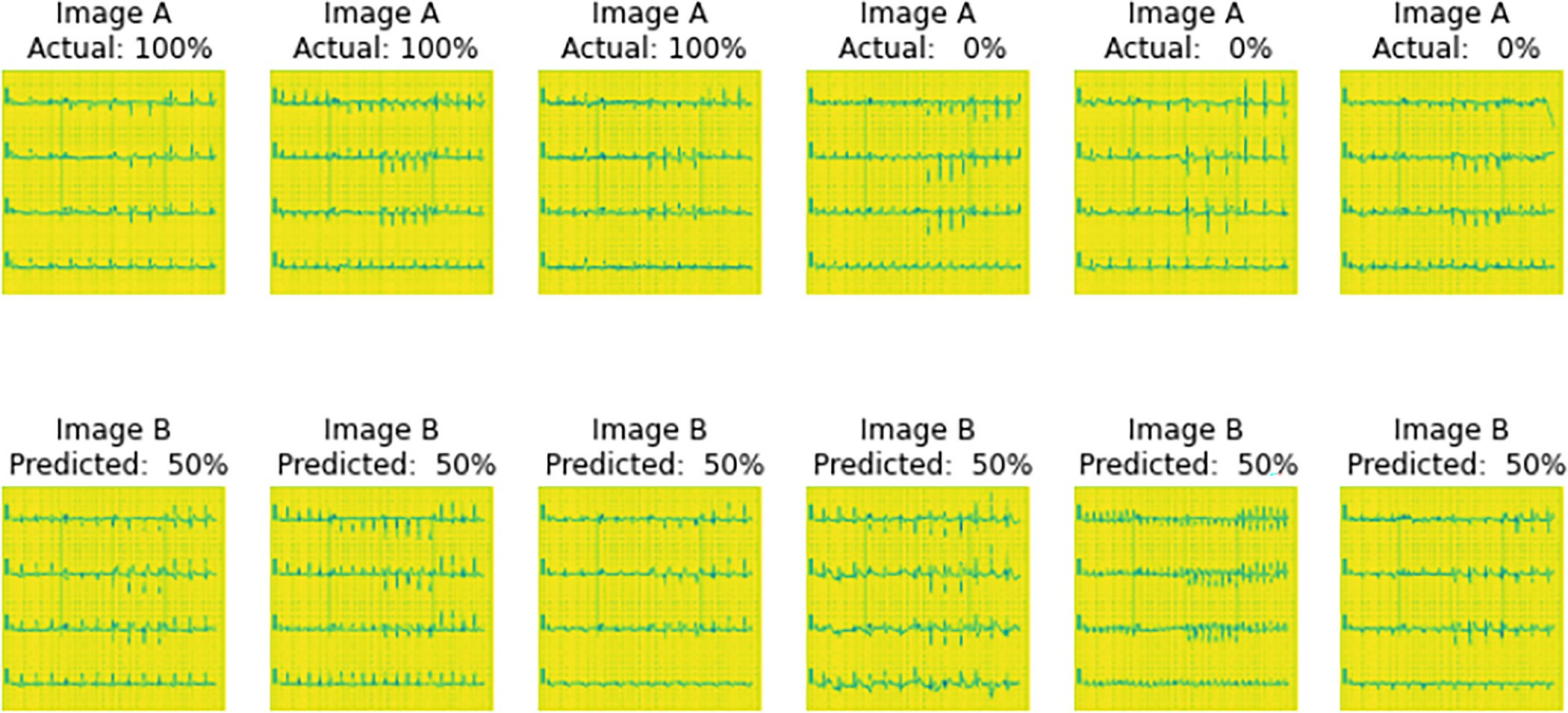
Sample images that were feed to untrained model.

**Fig 7:**
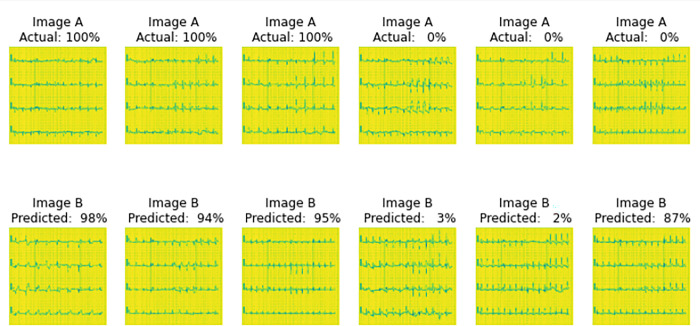
Sample images that were fed to the trained model.

The image dataset was taken from “ECG Images Dataset of Cardiac Patients”, Mendeley Data [8]. The pre-processing included cropping, re-sizing, background noise removal, etc. The dataset contains three different classes namely Abnormal heartbeat—233 images, Normal—284 images, and Myocardial Infarction—411 images each of size 256*256 (as shown in Table 1, Figs 3–7). The images were randomly shuffled and were split into train 70%, validation 20% and test 10% of whole. Further details on distribution include:

**Table 1:** Class distribution of dataset.

	Abnormal Heartbeat	Normal Heartbeat	Myocardial Infarction
**Train Dataset**	163	198	287
**Validation Dataset**	46	56	82
**Test Dataset**	24	30	42

Since, the total availability of the images for training purposes of less, the Siamese network classifier was used to mitigate the problem of a smaller number of images and class imbalance. Fig 4 represents the sample ECG image that is used for the purpose of training.

Fig 5 represents the images that will feed to the untrained Siamese model that is, Image A and Image B of the same column with a similarity of 100% resemble they belong to the same class and the one with 0% represents they belong to a different class. Image A and Image B of the same column are fed to the model represented in Fig 5 at the same time for the purpose of comparison and similarity.

#### Before training

Fig 6 represents the image A and image B of the same column were given for the untrained model (Figs 4–7) to predict the similarity between the images. The actual percentage represents how close/ same class the Image A and B are in actual, the predicted percentage tells the confidence of the model (Figs 4–7) how close/same class Image A and B are. It clearly shows the model is confused.

### Parameters for training

Table 2 below shows the hyperparameters used in the model.

**Table 2:** Model parameters.

Hyperparameters	Values
Number of trainable parameters	15249952
Optimizer	Adam
Output layer activation function	Sigmoid
Hidden layer activation function	ReLu
Batch Size	32
Number of epochs	20
Loss Function	Binary Cross Entropy

#### After training

Fig 7, shows that after training the Siamese network model, images were made to predict the similarity score between image A and Image B of the same column. The actual 100% on Image A signifies that both Image A and B belong to the same class and they are similar in Actual while the predicted on image B signifies the model prediction score of the similarity between Image A and B.

### b) Parameter tuning

Optimizing the Siamese Network Model for detecting Myocardial Infarction (MI) from ECG signals required careful adjustment of several key settings.

*Learning Rate*:

Tested rates: 0.001, 0.0001, and 0.00001.

The rate of 0.0001 balanced speed and accuracy well.

Higher rates led to quick but often suboptimal convergence.

Lower rates made training too slow.

*Batch Size*:

Tested sizes: 16, 32, and 64.

Size of 32 was optimal for stability and efficiency.

Smaller sizes caused noisy updates.

Larger sizes required more memory without much performance gain.

*Number of Convolutional Layers*:

Tried 2, 3, and 4 layers.

Three layers captured necessary features best without overfitting.

More layers added unnecessary complexity.

*Dropout Rate*:

Tested rates: 0.1, 0.3, and 0.5.

The rate of 0.1 prevented overfitting while maintaining performance.

Higher rates led to underfitting.

*Activation Functions*:

ReLU was used for hidden layers.

Sigmoid was used for the output layer.

ReLU helped with faster convergence.

Sigmoid was suitable for binary classification.

*Optimizer*:

Adam optimizer was chosen for its adaptive learning rate.

Combined benefits of AdaGrad and RMSProp, making it effective for sparse gradients.

## 5. Results

**Fig 8:**
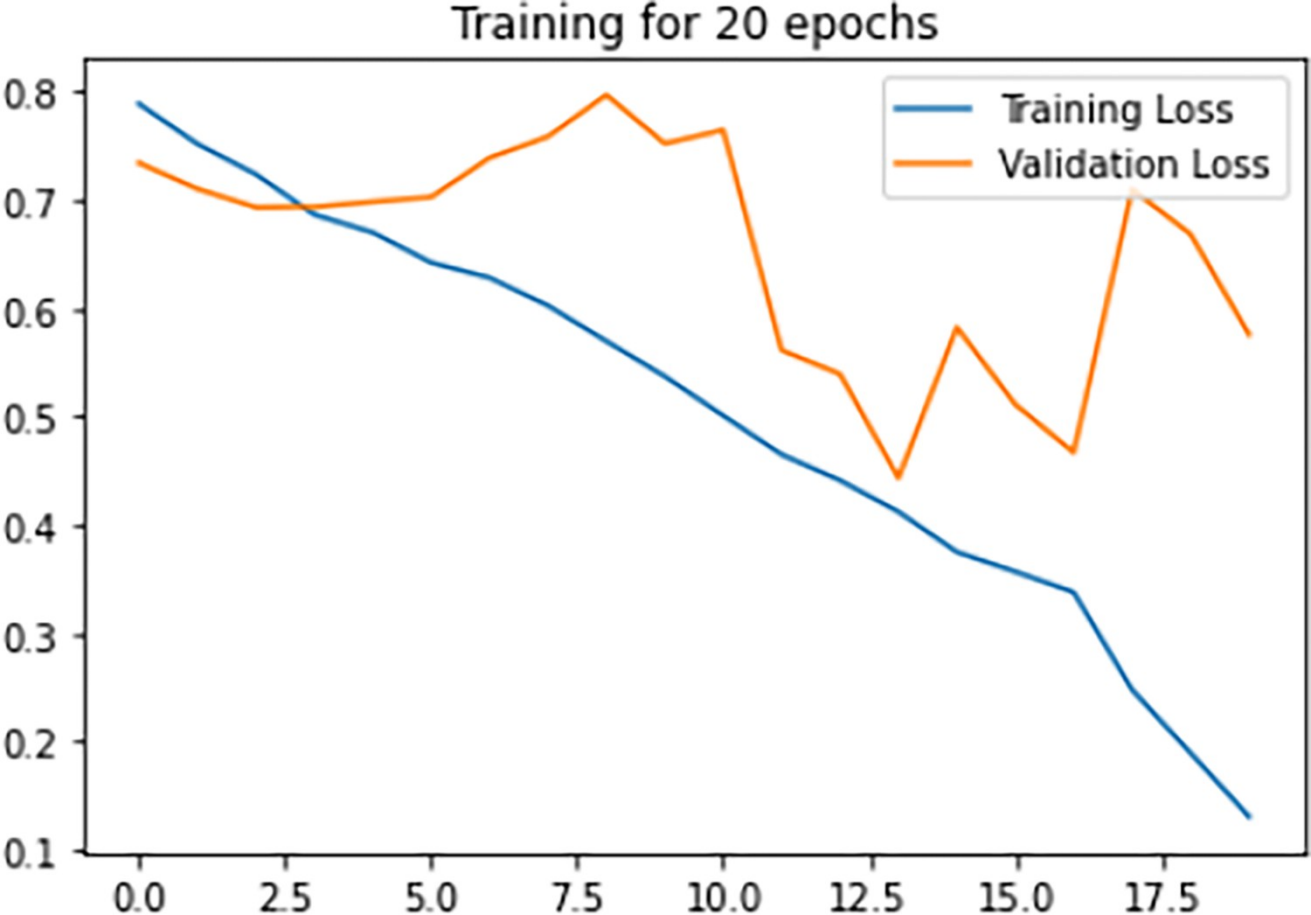
Loss vs epochs during training phase (20 epochs).

Fig 8, signify the training loss, validation loss vs epochs of the Siamese network model during the training phase. Training loss is evaluated on the training Images while validation loss is evaluated for validation images for every epoch. The lesser the training loss and validation loss, the better the performance of the classification of Images. From the below graph, it is clear that the model was clearly in the direction of overfitting which can be seen from the curve of validation loss as it was pumping up more towards loss than reducing the validation loss.

Fig 9 signifies the training accuracy, validation accuracy vs epochs of the Siamese network model (Figs 4–7) during the training phase. Training accuracy is evaluated on the training Images while validation accuracy is evaluated for validation images for every epoch. The higher the training accuracy and validation accuracy, the better the performance of the classification of Images. Similarly, Figs 10–17 represents the training accuracy for varied epochs.

**Fig 9:**
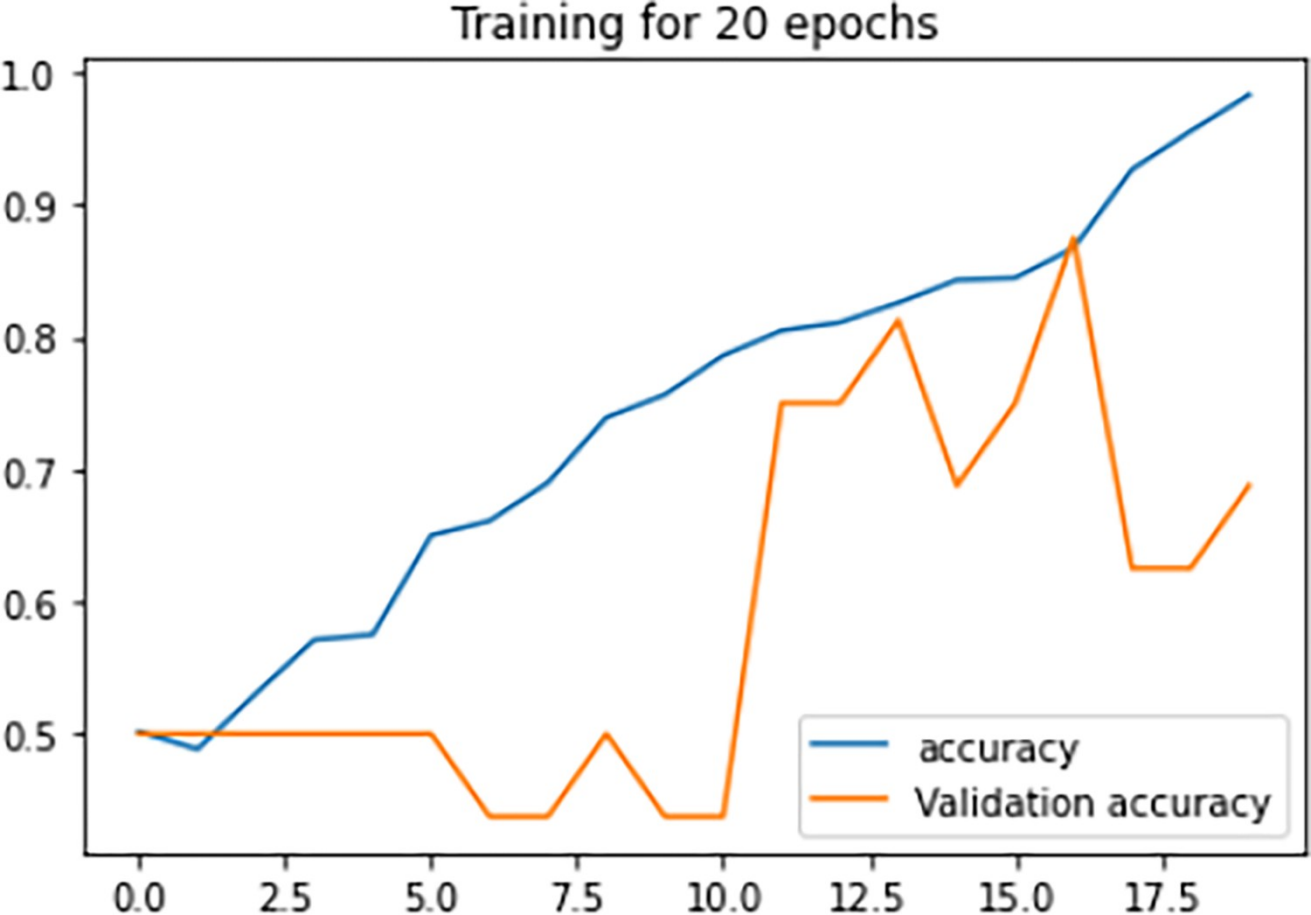
Accuracy vs epochs during training phase (20 epochs).

**Fig 10:**
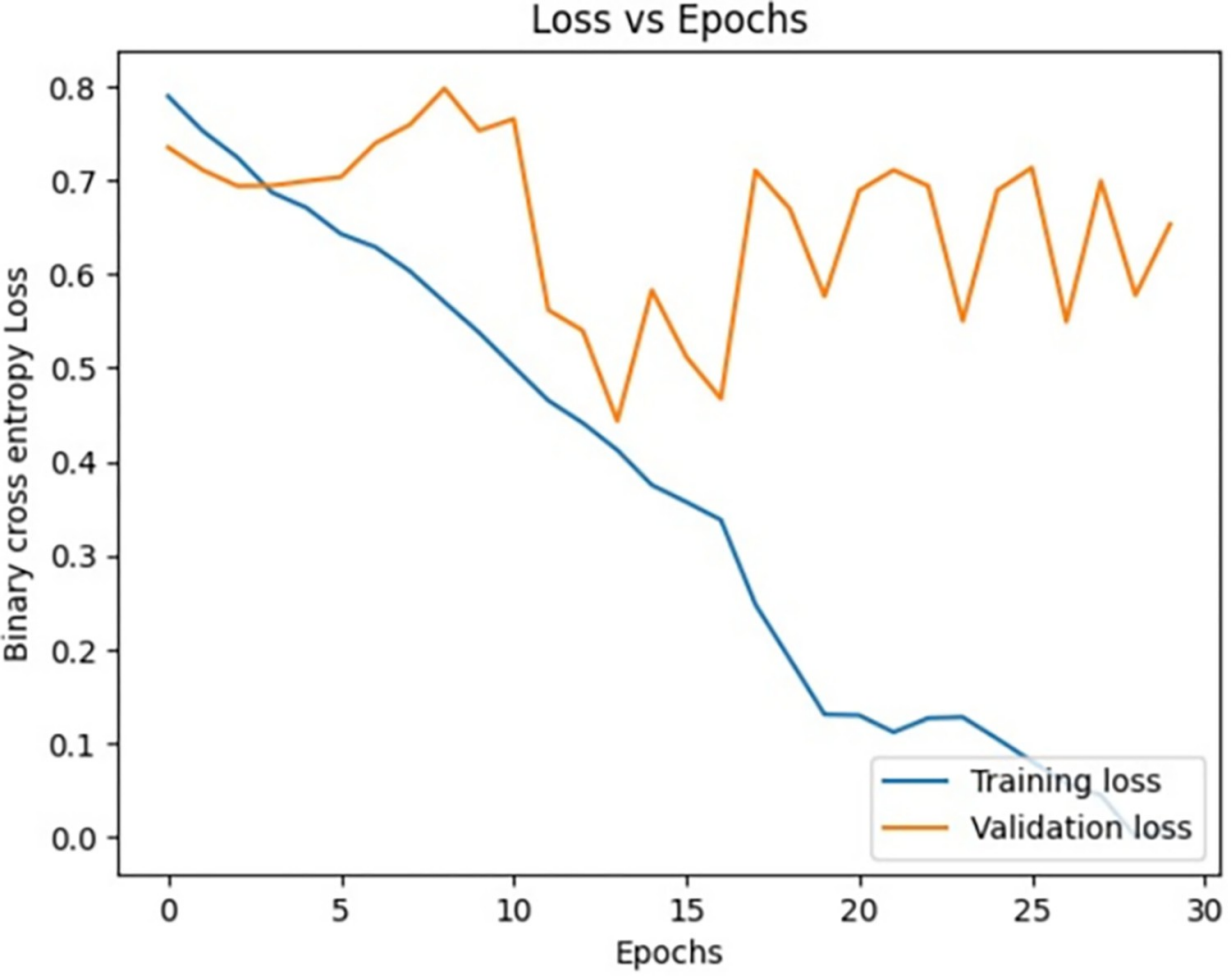
Loss vs epochs during training phase (30 epochs).

**Fig 11:**
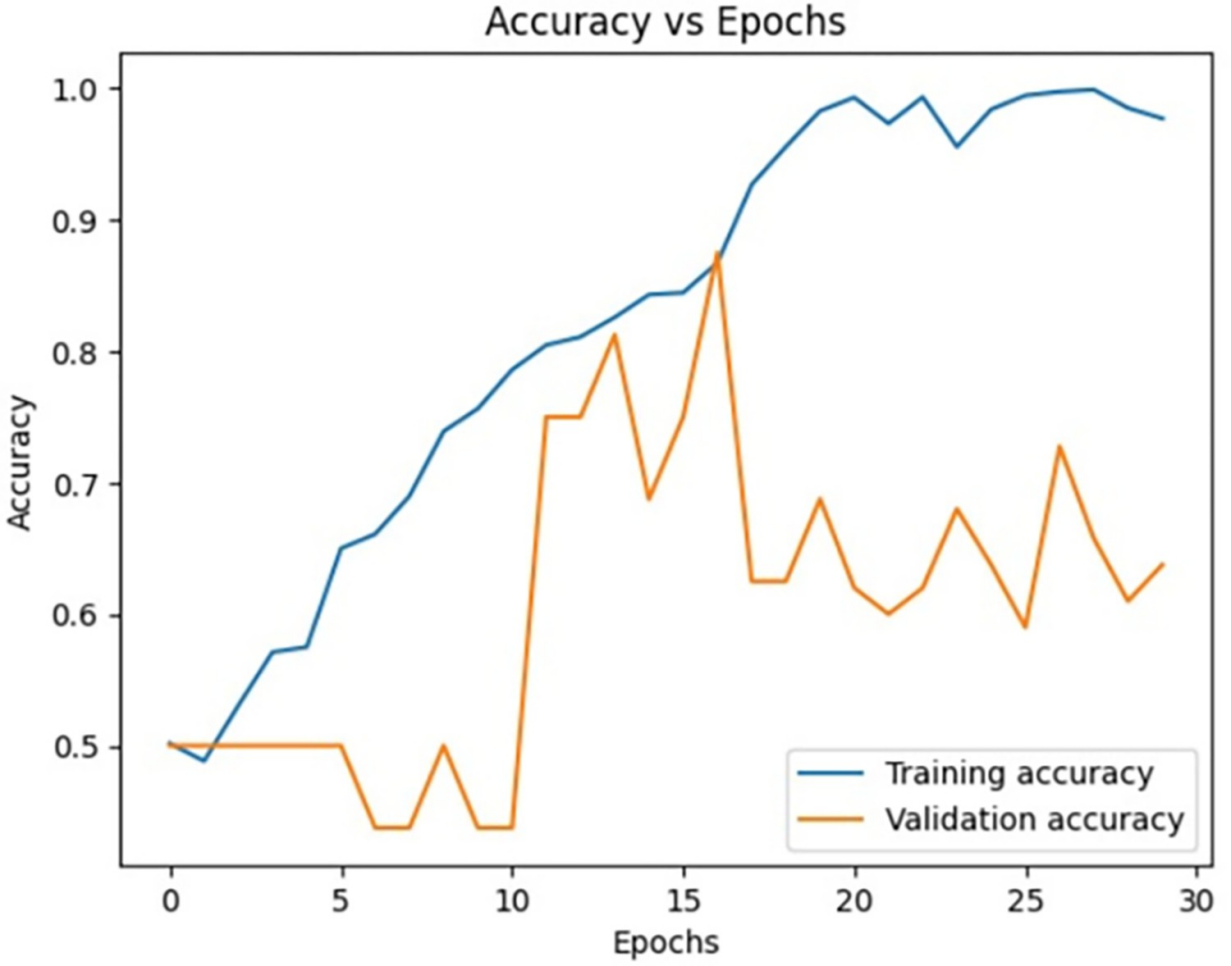
Accuracy vs epochs during training phase(30 epochs).

**Fig 12:**
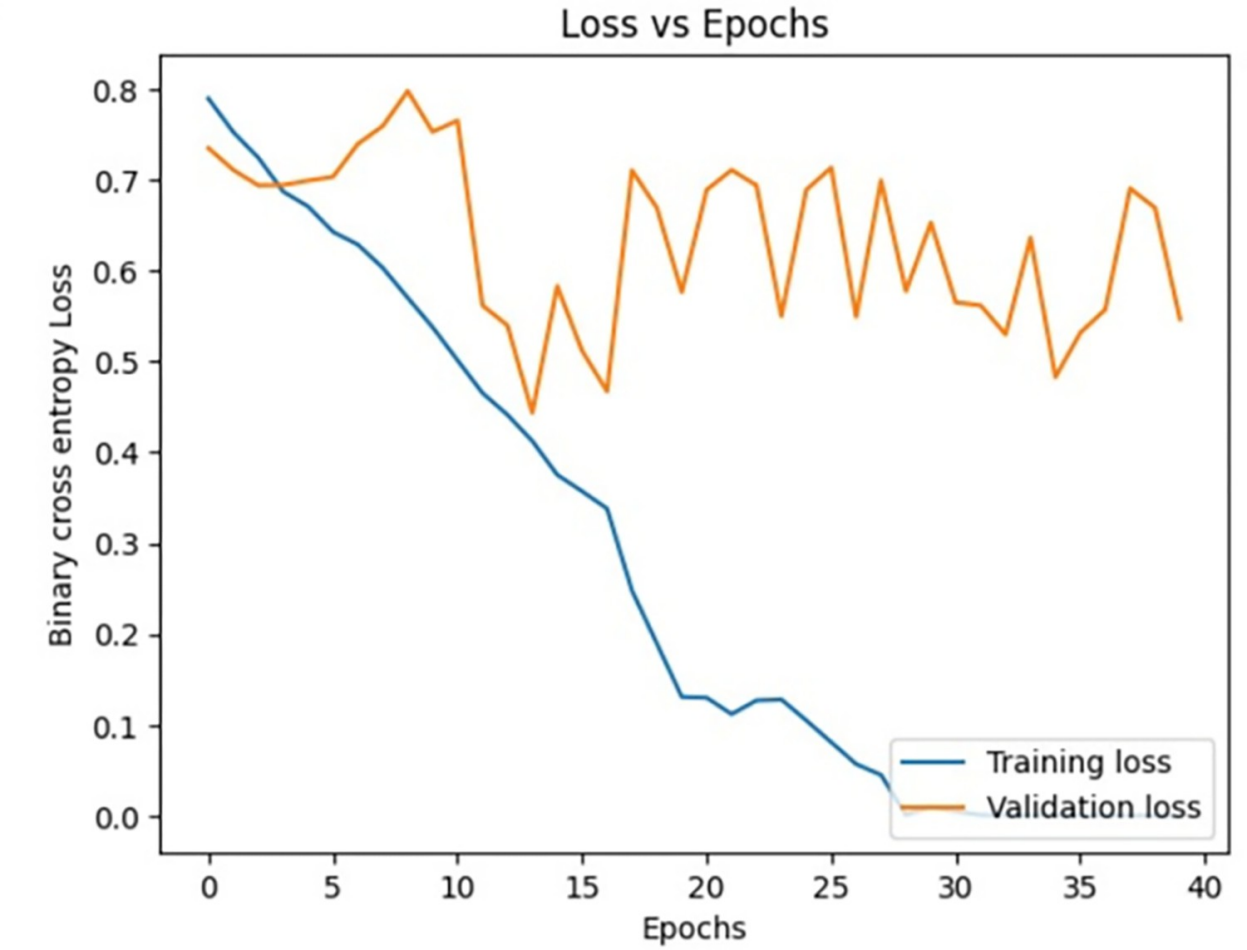
Loss vs epochs during training phase(40 epochs).

**Fig 13:**
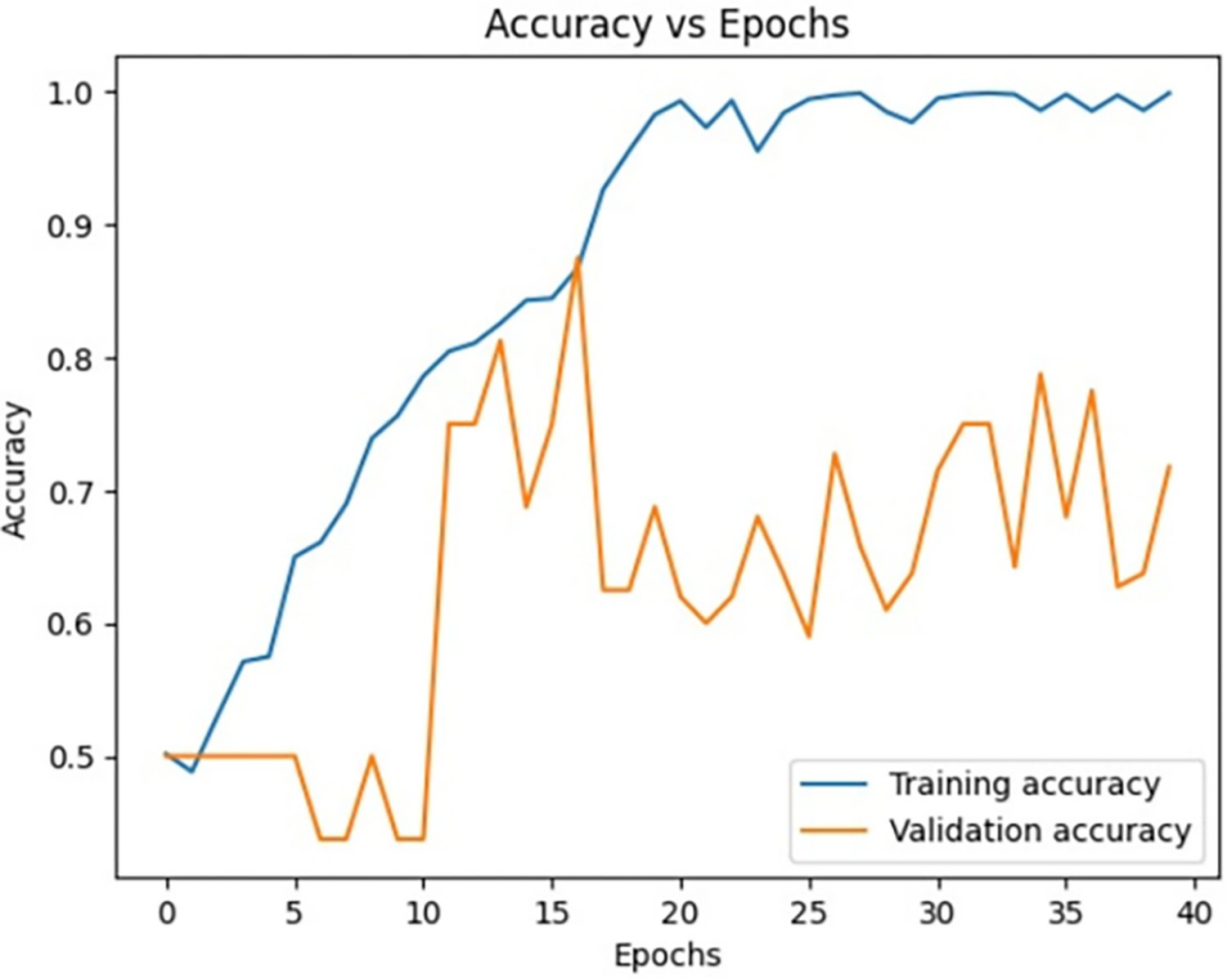
Accuracy vs epochs during training phase(40 epochs).

**Fig 14:**
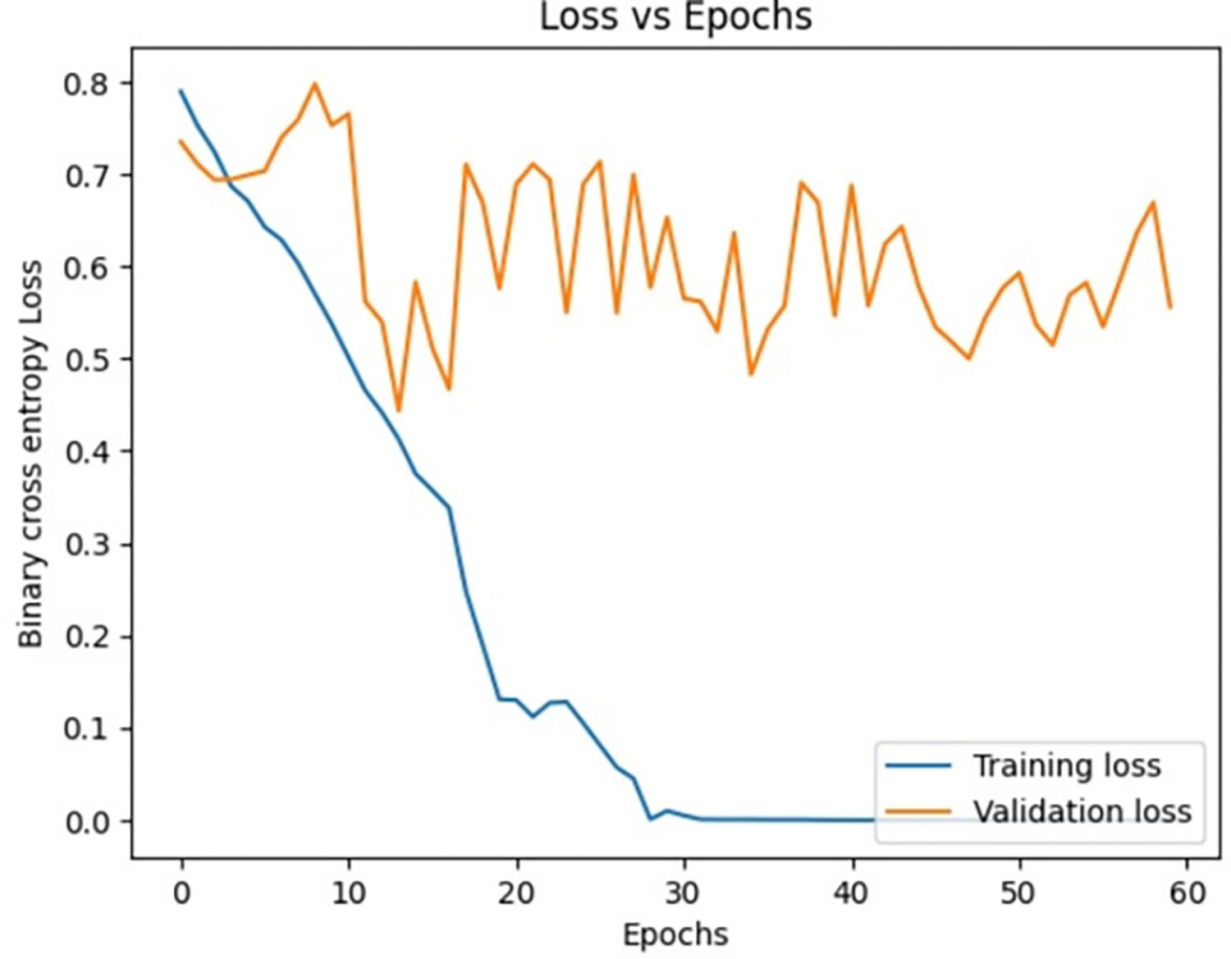
Loss vs epochs during training phase(60 epochs).

**Fig 15:**
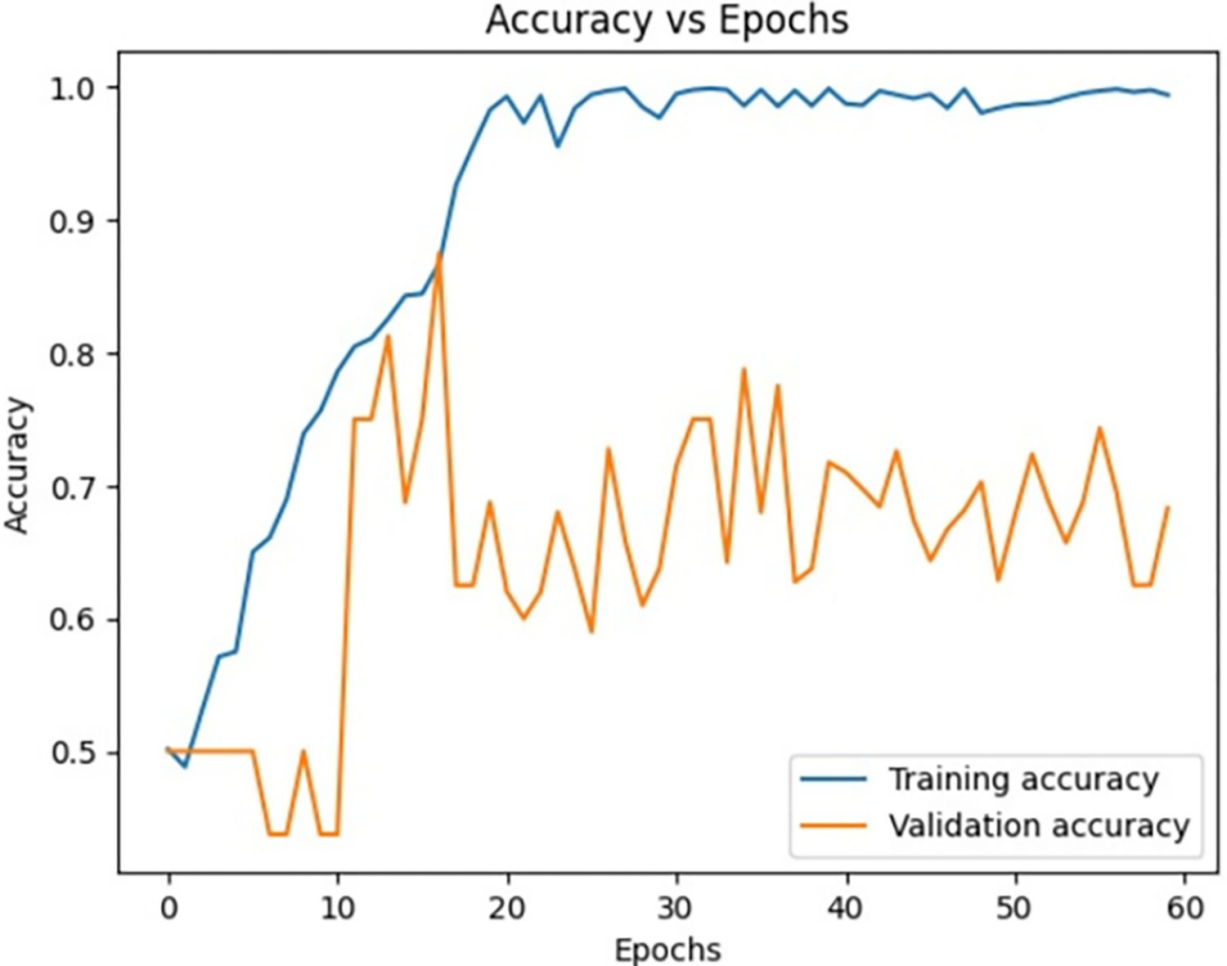
Accuracy vs epochs during training phase(60 epochs).

**Fig 16:**
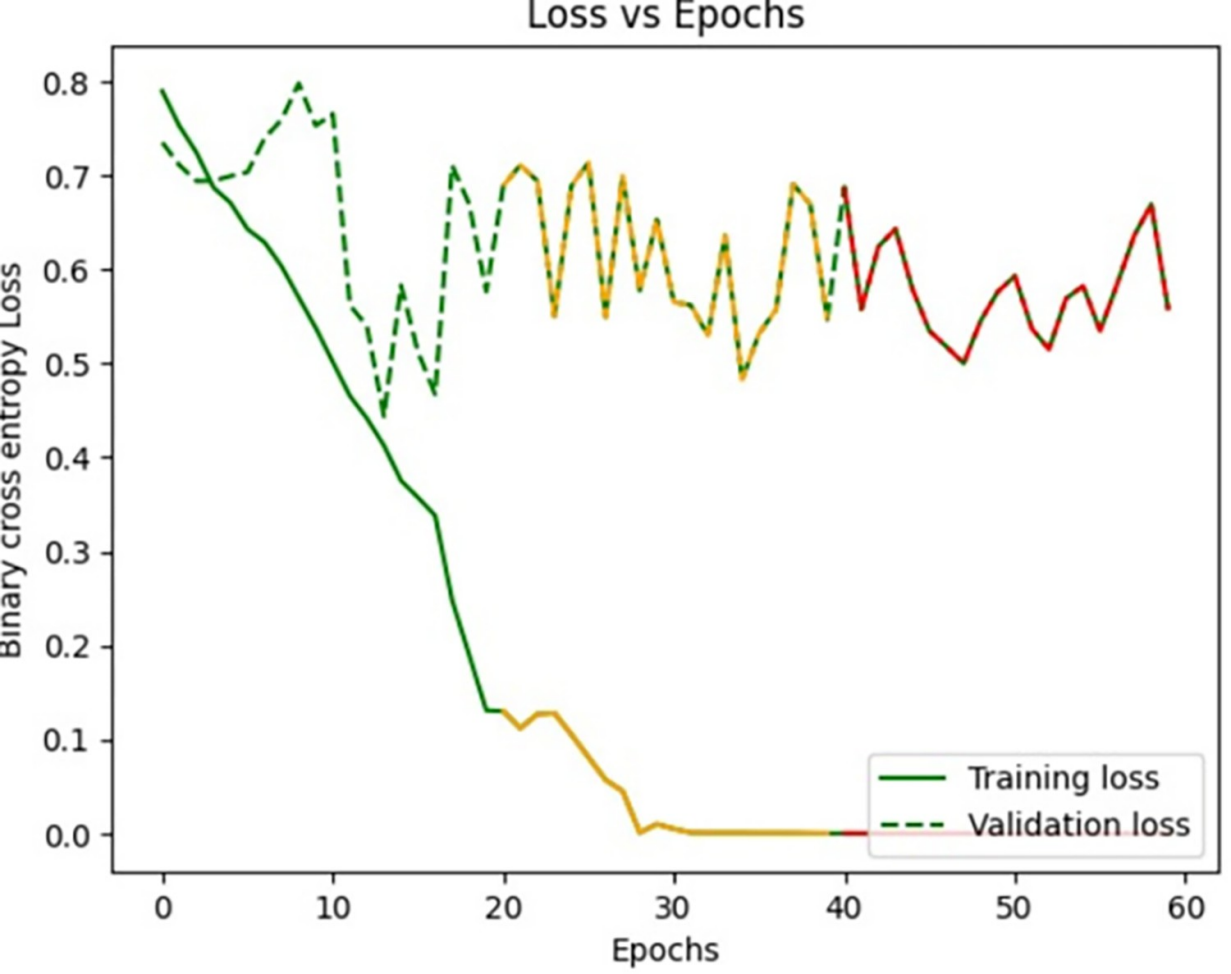
Loss vs epochs during training phase (Overall).

**Fig 17:**
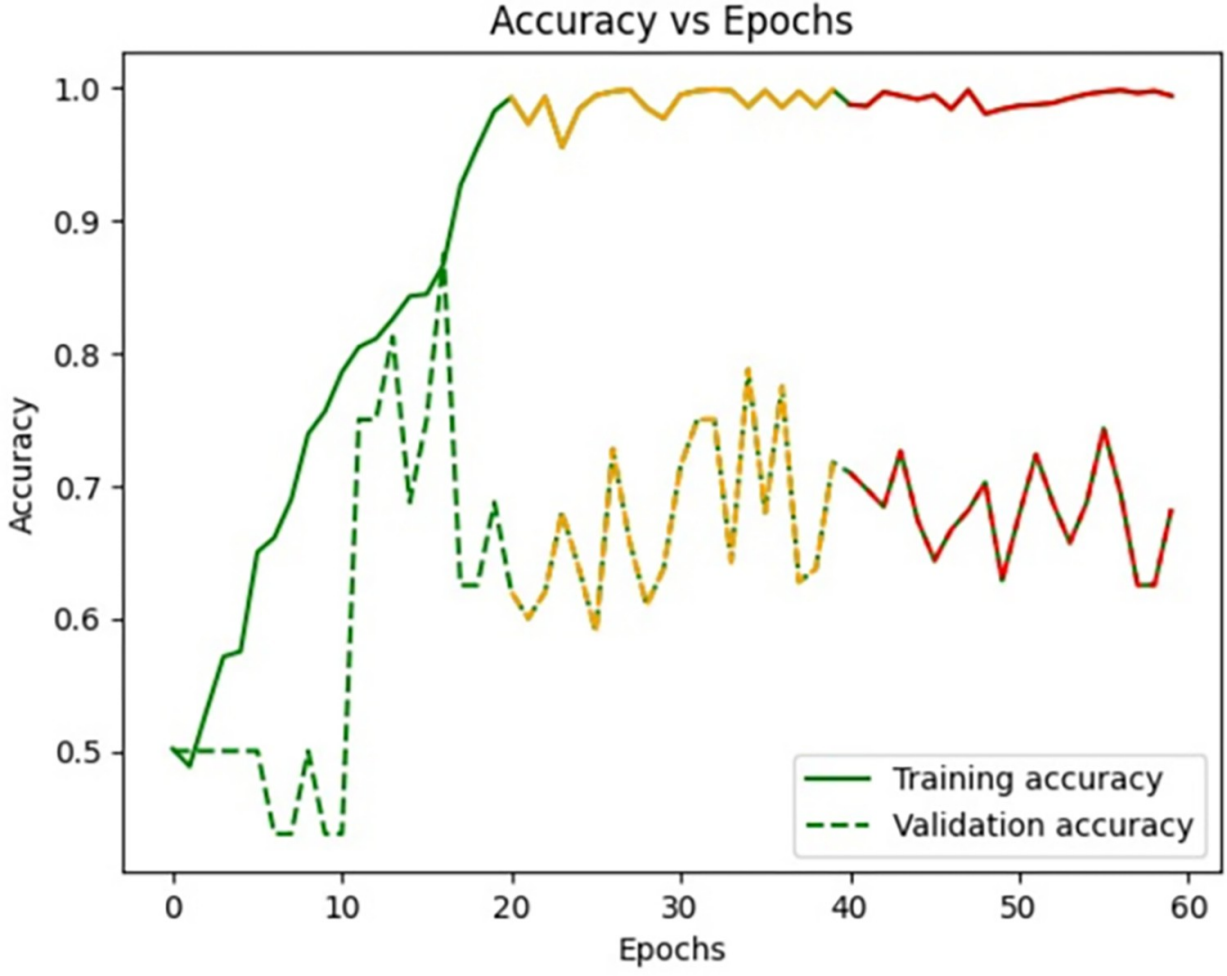
Accuracy vs epochs during training phase(Overall).

### a) Performance definition

In the context of evaluating a machine learning model, accuracy measures the proportion of correctly classified instances out of the total instances. Precision indicates the proportion of true positive predictions among all positive predictions made by the model, emphasizing the model’s ability to avoid false positives. Recall, on the other hand, measures the proportion of true positive predictions among all actual positive instances in the dataset, focusing on the model’s ability to capture all positive instances. The F1 score, a harmonic mean of precision and recall, provides a balanced assessment of the model’s performance. Specificity measures the proportion of true negative predictions among all actual negative instances, while the false negative rate quantifies the proportion of actual positive instances incorrectly classified as negative by the model. Conversely, the false positive rate calculates the proportion of actual negative instances incorrectly classified as positive. These metrics collectively offer insights into different aspects of the model’s performance, aiding in its optimization and interpretation. The terms TP, TN, FP, and FN in formulas denote the count of true positive predictions, true negative predictions, false positive predictions, and false negative predictions, respectively as show in Eqs (2)–(8).


$$Accuracy=\frac{\left(TP+TN\right)}{\left(TP+FP+TN+FN\right)}$$ (2)

$$Precision=\frac{TP}{\left(TP+FP\right)}$$ (3)

$$Recall=\frac{TP}{\left(TP+FN\right)}$$ (4)

$$Specificity=\frac{TN}{\left(TN+FP\right)}$$ (5)

$$F1\;Score=\frac{2*(Precision*Recall)}{\left(Precision+Recall\right)}$$ (6)

$$False\;Negative\;Rate\;(FNR)=1-\frac{TP}{\left(TP+FN\right)}$$ (7)

$$False\;Positive\;Rate\;(FPR)=\frac{FP}{\left(FP+TN\right)}$$ (8)

### b) Test result on training dataset

The Table 3 represents the performance of the model of the training Images. For the class Abnormal heartbeat, there were 171 images used for training, the F1 score obtained to classify the abnormal heartbeat images is 0.98, recall is 0.95 and precision is 1.0. For the class Normal, there were 202 images used for training, the F1 score obtained to classify the Normal images is 0.98, recall is 0.97 and precision is 0.99. For the class Myocardial Infarction, there were 275 images used for training, the F1 score obtained to classify the Myocardial Infarction images is 0.98, recall as 1 and precision as 0.96.

**Table 3:** Performance proposed model of training images.

	Precision	Recall	F1 score	Support	Specificity	FNR	FPR
**Abnormal Heartbeat**	1.00	0.95	0.98	171	1.00	0.05	0
**Normal**	0.99	0.97	0.98	202	0.99	0.03	0.004
**Myocardial Infarction**	0.96	1.00	0.98	275	0.97	0	0.03

Table 4 represents the overall accuracy for the training images i.e 648 images is 0.98.

**Table 4:** The overall accuracy for the training images.

**Accuracy(%)**			0.98	648
**Macro Avg(%)**	0.98	0.97	0.98	648
**Weighted Avg(%)**	0.98	0.98	0.98	648

### c) Confusion matrix on training dataset

**Fig 18:**
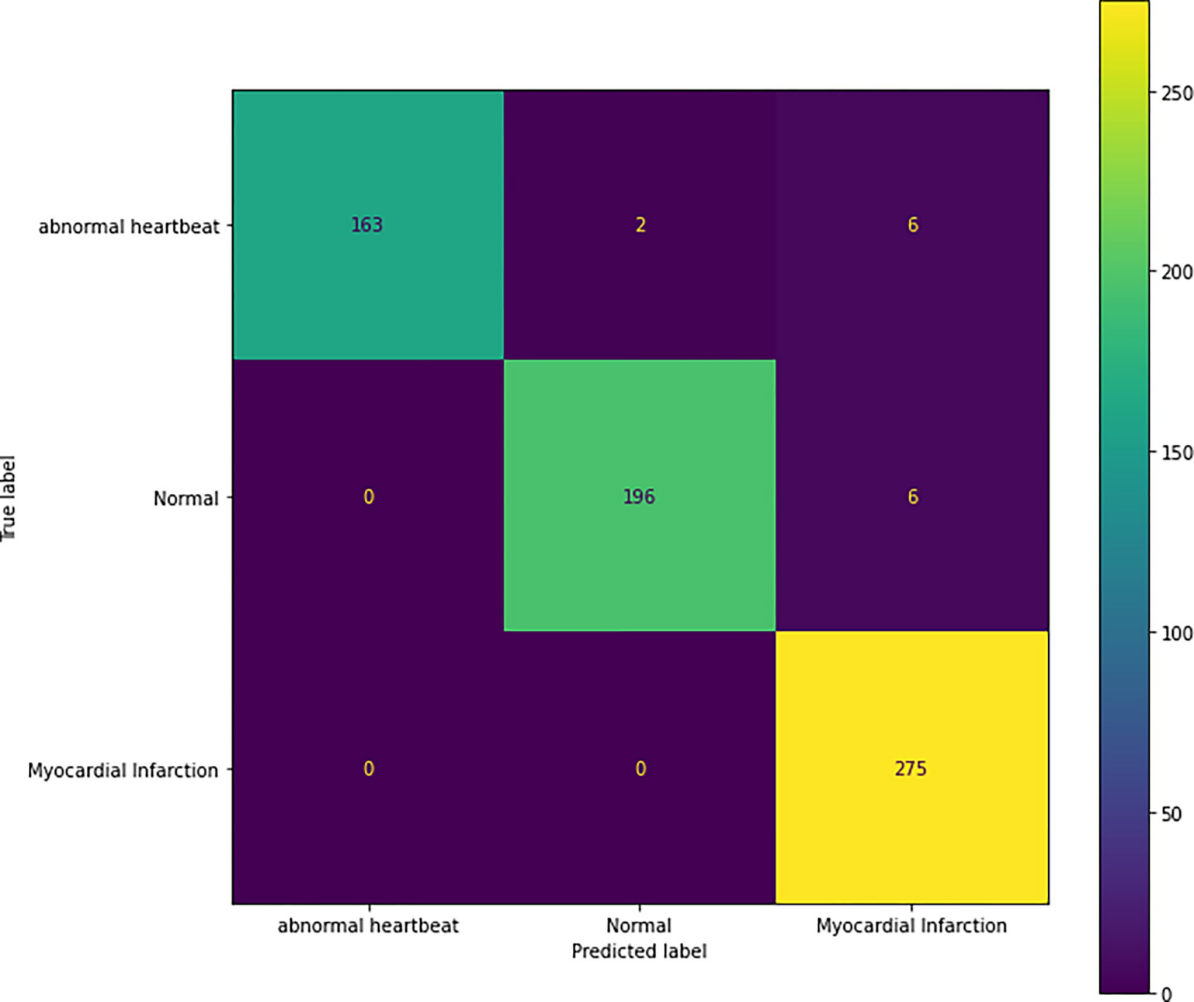
Confusion matrix on training image.

Fig 18, represents the prediction done on training image and tells the number of correct and incorrect prediction done for each class. For Abnormal heartbeat, out of 171 images, the model classified 163 of them correctly. For Normal images, out of 202, the model classified 196 of them correctly and for Myocardial Infarction out of 275 images, it classified 275 of them correctly.

The Table 5 represents the performance of model of the training Images. For the class Abnormal heartbeat, there were 63 images used for validation, the F1 score obtained to classify the abnormal heartbeat images is 0.84, recall is 0.73 and precision is 1.0. For the class Normal, there were 42 images used for validation, the F1 score obtained to classify the Normal images is 0.71, recall is 0.83 and precision is 0.62. For the class Myocardial Infarction, there were 79 images used for validation, the F1 score obtained to classify the Myocardial Infarction images is 0.88, recall as 0.9 and precision as 0.87.

**Table 5:** Performance model for training images.

	Precision(%)	Recall(%)	F1 score(%)	Support(%)	Specificity	FNR	FPR
**Abnormal Heartbeat**	1.00	0.73	0.84	63	1.00	0.27	0
**Normal**	0.62	0.83	0.71	42	0.87	0.17	0.13
**Myocardial Infarction**	0.87	0.90	0.88	79	0.90	0.10	0.09

Table 6 represents the overall accuracy for the images i.e 184 images is 0.83.

**Table 6:** The overall accuracy for the images.

**Accuracy**	0.83	184
**Macro Avg**	0.81	184
**Weighted Avg**	0.83	184

### d) Confusion matrix on validation dataset

**Fig 19:**
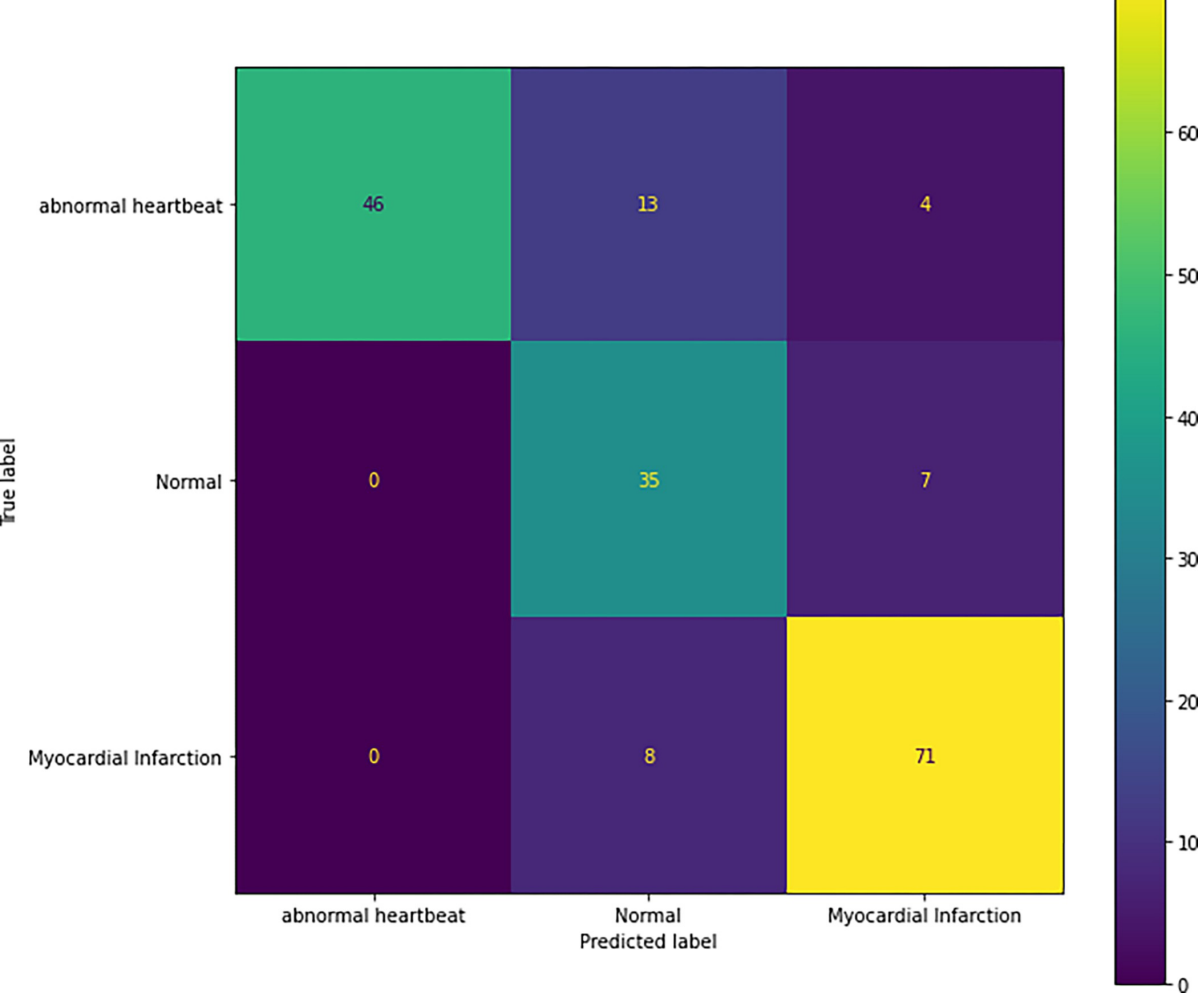
Confusion matrix on validation image.

Fig 19 represents the prediction done on the validation image and tells the number of correct and incorrect prediction done for each class. For Abnormal heartbeat, out of 63 images, the model classified 46 of them correctly. For Normal images, out of 42, the model classified 35 of them correctly and for Myocardial Infarction out of 79 images, it classified 78 of them correctly.

The Table 7 represents the performance of model (Fig 3) of the training Images. For the class Abnormal heartbeat, there were 30 images used for testing, the F1 score obtained to classify the abnormal heartbeat images is 0.89, recall is 0.80 and precision is 1.0. For the class Normal, there were 28 images used for testing, the F1 score obtained to classify the Normal images is 0.90, recall is 0.93 and precision is 0.87. For the class Myocardial Infarction, there were 38 images used for training, the F1 score obtained to classify the Myocardial Infarction images is 0.90, recall as 0.95 and precision as 0.86.

**Table 7:** Performance model of training images.

	Precision	Recall	F1-Score	Support	Specificity	FNR	FPR
Abnormal heartbeat	1.00	0.80	0.89	30	1.00	0.20	0
Normal	0.87	0.93	0.90	28	0.94	0.07	0.05
Myocardial Infarction	0.86	0.95	0.90	38	0.91	0.05	0.09

Table 8 represents the overall accuracy for the images i.e 96 images is 0.9.

**Table 8:** The overall accuracy for the images.

**Accuracy (%)**	0.9	96
**Macro Avg(%)**	0.9	96
**Weighted Avg(%)**	0.9	96

### e) Confusion matrix on test dataset

**Fig 20:**
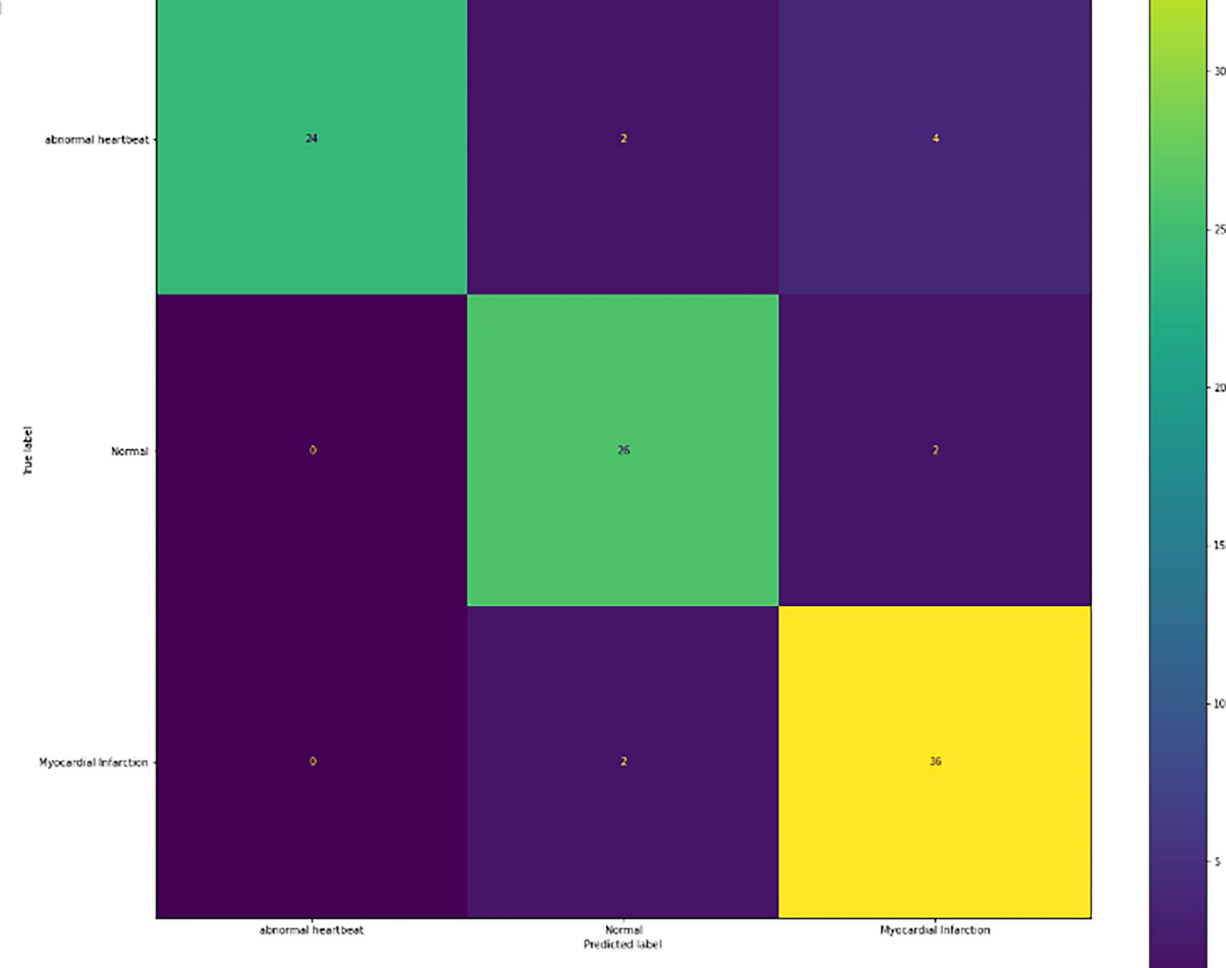
Confusion matrix on test image.

The Fig 20 represents the prediction done on a test image and tells the number of correct and incorrect prediction done for each class. For Abnormal heartbeat, out of 30 images, the model classified 24 of them correctly. For Normal images, out of 28, the model classified 26 of them correctly and for Myocardial Infarction out of 38 images, it classified 36 of them correctly.

From the above results we can see that the F1 score was the same for all the 3 categories in training dataset i.e 0.98 & accuracy to be 98%. The F1 score for validation dataset achieved was higher for myocardial infarction i.e 0.88, abnormal heartbeat i.e 0.84 & normal i.e 0.71 & accuracy to be 0.83. The F1 score for test dataset achieved overall remained same for Normal & Myocardial Infraction i.e 0.9 & Abnormal heartbeat to be 0.89 & accuracy to be 0.9.

In recent years, with the rise of deep learning and machine learning methods, the general detection and diagnosis of cardiovascular diseases and specific Myocardial Infarction (MI) in ECG signals have improved. The Siamese Network Model proposed in this work, and its approach to handling class imbalance, limited data, creating novelties compared to the mainstream models such as hybrid networks of CNN-LSTM, DenseNets, Generative Adversarial Networks, etc. The first and foremost of the benefits of the Siamese Network Model is that it is scalable. Most traditional models are overly reliant on extensive pre-processing and hand-crafted feature extraction, which increases the complexity and computational cost of feature engineering. The Siamese Network directly processes raw ECG images, effectively reducing complexity and the computational burden that feature engineering puts on the overall scalability for deployment in diverse clinical settings with varying levels of computational resources.

The model, Siamese Network Model, is flexible in its design while assessing interoperability with different datasets and the varying quality of the ECG signals. It can learn from pairs of images and concentrate on similarity and dissimilarity of these pairs. Hence, making it generalize well across different populations and clinical environments. In contrast, many state-of-the-art models require specific adjustments and fine-tuning for a different dataset, often leading to less-than-ideal generalization. However, regulatory compliance in deploying AI models in healthcare should be very important to consider. The Siamese Network Model is simple and transparent, supporting the model characteristics that would allow for a more straightforward regulatory approval than models like GANs, which perform really well but often lack interpretability in a way that makes them hard to fit against stringent regulatory standards. This, in addition to its high performance metrics, is enabled because of the plain architecture in the Siamese Network, meaning all that would work well against the regulatory requirements and guarantee its embedding into clinical workflows.

However, the bottleneck of scalability and interoperability is still there. High computational power for training acts as a barrier, particularly for resource-constrained environments; optimization should thus be extended further to make the model accessible. Although promising, the robustness and applicability across healthcare settings from such innovative approaches need extensive validation on much larger multi-center datasets. Conclusively, the Siamese Network Model offers great promise as an alternative to current state-of-the-art approaches and includes key important features pertaining to scalability and interoperability, meeting critical regulatory compliance. All these developments make it more practical in a general sense for implementation on a broad scale for early MI detection.

### f) Research gaps

Further validation is needed on larger datasets but can be improved by pointing out specific research gaps and future directions. The current dataset includes 928 ECG images, which might not capture all the variability that exists in different populations and clinical settings. Such future work should enlarge their datasets to include a diverse patient demographic with comorbidities and clinical conditions through collaborations with multiple medical institutions. The study based on static images of ECGs is missing important information regarding the temporal dynamics. Adding continuous signals of ECGs and temporal deep learning models, including but not limited to RNNs or LSTMs, would enhance detection of subtle patterns. Deep learning models often act as black boxes, resulting in poor clinical adoption without interpretability. Techniques have to be developed further towards interpretability, for example, Grad-CAM and SHAP, which are necessary for regulatory compliance.

To the model developed in the present work, to be able to tune real-time monitoring, the next stage would be integrating it within a real-time monitoring system, for example, wearable devices, to provide continuous analysis and prompt alerting. Although the Siamese Network Model does take into account class imbalance, rare conditions are still hard to tackle. Advanced techniques, such as GANs or cost-sensitive learning, can be explored for this task, and the models developed will put more focus on robustness in rare scenarios. Lastly, the heavy amount of ECG data in the study conducted means that it might overlook information described by different diagnostic modalities. Multi-modal data integration could provide a comprehensive view of cardiac health for diagnostic accuracy, therefore improving information on cardiovascular diseases by adding in echocardiography, MRI, and patient history. In conclusion, we addressed the research gap by creating a Siamese Network Model that manages class imbalance and limited data, processes raw ECG images directly, and enhances the accuracy of MI detection.

## 6. Discussion

The primary objective of this work was to establish and assess a Siamese Network Model for the early detection and classification of Myocardial Infarction from ECG signals. The achieved results with the suggested model provide an effective rate of 98% in classification accuracy. This section details the implications of such findings, compares them with previous studies, and finally discusses the strengths and limitations of the current approach.

### Implications of findings

- The high accuracy accomplished with the Siamese network model represents a significant improvement in automated MI detection using ECG signals.
- Conventional methods, including manual interpretation of ECG signals, are likely to be biased by human errors and observer bias.
- The current model overcomes these limitations and presents an efficient and automatic way.
- Its competence toward handling class imbalance and limited data further renders it more practical for real-world clinical settings, where data availability is one of the major challenges.

### Comparison with previous studies

- Comparing our results with existing literature highlights the advancements made by our approach.
- For example, using a hybrid CNN-LSTM model, Rai *et al*. achieved an accuracy of 95.5%. However, it was still effective and needed much pre-processing, as well as hand-crafted features.
- Similarly, Jahmunah et al. achieved an accuracy of 88.7% through a DenseNet model, which, although it proved to be robust, was complex in terms of network architecture and required significant computational resources.
- The proposed Siamese Network Model surpasses all these accuracies and, in addition, has the advantage of working directly with raw ECG images.
- It reduces the very high demand for pre-processing and feature extraction, makes the job much simpler, and, thus, makes the model more implementable in various clinical environments.

### Strengths of the proposed model

- Learning the Siamese network model from the pair of images focused on their similarity and dissimilarity is one of the critical strengths.
- Inherently, this technique will solve the imbalance issue present in almost all the medical datasets.
- The traditional models fail sometimes to capture the features of the underpopulated classes, further leading to biased outcomes.
- Our model therefore ensures learning robust features from limited data and enhances generalizability through its pair-based learning mechanism.
- Another major strength of this model is in the architecture itself.
- Making use of identical subnetworks to extract features from different inputs guarantees consistency during the learning process.
- For this purpose, convolutional layers are used to capture spatial features, while dense layers are used for classification, resulting in the appropriate balance in feature extraction.

### Limitations and challenges

- Despite its promising results, this study still has some limitations.
- First, the model developed would require large computational resources during the training phase, thus rendering the model applicable in a selective range of resource-endowed applications.
- The model is working efficiently at inference time, but the training phase could be optimized regarding resource consumption.
- Secondly, the model’s performance was evaluated on a relatively small and specific dataset.
- Although the results are encouraging, further validation on larger and more diverse datasets is necessary to confirm the model’s robustness and applicability across different populations and clinical settings.
- Third, static images of ECG traces were used, and not the original sequence data.
- While a lot can be inferred from such images, further integration of temporal information—with continuous signals—would likely make the model perform better.
- Future work could investigate the inclusion of RNNs or LSTM networks to learn dynamic temporal dependencies within ECG sequences.

### Future directions

- Research should now aim to remove the limitations that this study discovered.
- A main area will be developing the model architecture and training process to reduce computational needs.
- It would also be important to carry out further validation of the model on larger, multi-center datasets in order to learn about its generalizability.
- Some other interesting studies can be done on incorporating temporal dynamics into the ECG data, using advanced neural network architectures for a deep analysis of cardiac conditions.
- The Siamese network model is, therefore, a big leap in the automation of myocardial infarction detection based on ECG signals.
- The model provides a robust solution to solve the issue of class imbalance and data limitation; hence, it promises sustainability in diagnosis for early treatment to improve the outcomes of the patients and reduce the burden on healthcare providers.

## 7. Ablation study

In the machine learning domain, and more specifically in deep learning research, the ablation study is one of the most important studies to go through to understand the impact of each part and hyperparameters on the performance of the model. In this section, we go further into the different parts of our Siamese Network Model (SNN) to evaluate its efficacy in detecting Myocardial Infarction (MI) from ECG signals. Systematic experiments were conducted by varying one parameter at a time while keeping all the others constant and observing how it affects the performance of the model.

### Hyperparameter tuning

One of the most important ingredients in building a neural network is the selection of hyperparameters. We fine-tuned several hyperparameters—among them, the learning rate, batch size, number of convolution layers, and dropout rate.

### Learning rate

This is the size of steps taken during optimization. For such parameters, different learning rates (0.001, 0.0001, and 0.00001) were experimented on. The one that best performed while keeping the balance between convergence speed and accuracy was 0.0001. A higher learning rate made the model converge quickly to some suboptimal solution, whereas a lower rate slowed down the convergence much.

### Batch size

The batch size would also affect the process stability of gradient descent. We experimented with various batch sizes, such as 16, 32, and 64. A batch size of 32 is optimum in attaining the right balance between computational efficiency and model performance. When a smaller batch size was used, the updates were too noisy, and with a larger batch size, too much memory was consumed at the cost of diminishing returns in increased performance.

### Number of convolutional layers

We changed the number of convolutional layers in the model—2, 3, and 4 layers—for finding the best depth. The three-layer model works best in this case because enough features were taken without overfitting. The model has a minimal margin for improvement after a certain number of layers. But with it, the chances of overfitting, as well as computational complexity, grow.

### Dropout rate

Among the various techniques for regularization is dropout, which decreases the likelihood of overfitting on training data. We tried the different rates of dropping out, like 0.1, 0.3, and 0.5. We found that in our scenario, a dropout rate of 0.1 was quite impressive; it saved the generalization capability for the model and balanced overfitting. Higher rates of dropout began to create underfitting, where the model did not learn major patterns.

### Feature extraction

The Siamese network is designed to have two copies of the identical subnetwork for processing input images. Each of them is independently responsible for feature extraction. We tested different settings of these subnetworks and their effects on performance.

#### Kernel size in convolution

From the network testing, we could conclude that the kernel size of 3x3 is optimal for capturing small details of the ECG images. Greater sizes of kernels captured more global features but at a cost of higher computational load and potential loss of finer details.

#### Pooling layers

These reduce the spatial dimensions of feature maps; thus, they help in reducing the computational load and help in controlling overfitting. The layers were experimented with during this project for max-pooling and average-pooling. The best performance was obtained from max-pooling; this is done by getting rid of unimportant features and saving the most important ones in a compressed file.

#### Model variants

To evaluate the strength and effectiveness of the Siamese Network Model, we compared it with some of the state-of-the-art popular architectures like Convolutional Neural Networks with Long Short-Term Memory networks and hybrid models of CNN-LSTMs. Traditional CNNs were also trained on the same dataset. Though the CNNs did well with accuracy, the class imbalance handling wasn’t that good for SNNs. The SNN outperformed the CNNs, which managed to learn the differences between pairs of similar and dissimilar signal samples and improved the generalization from fewer examples. In the case of LSTM and SNN, the LSTM, which is specifically designed for sequential data, found betterment on ECG signals. Even if LSTMs worked properly in the learning time dependencies of the dataset, it still got slow behind SNN when using image-based feature extractors.

#### Hybrid models

Some of the hybrid models used a combination of CNN and LSTM layers for the configuration. These models performed competitively, but both the training times and computational requirements were considerably more compared to the SNN. SNN was a simpler yet effective alternative.

### Ablation on training data

Due to the unavailability of data, here we evaluate our model in different cases of training data availability.

#### Data augmentation

We applied different augmentation approaches like rotation, scaling, and flipping. Further, data augmentation also increased model robustness, as shown through better class imbalance handling, in which accuracy of augmented data-trained models improved by 2 to 3% compared to their unbalanced data counterparts.

#### Class imbalance

The dataset is imbued with class imbalance, where more instances of MI exist in comparison with normal or abnormal heartbeats. To handle the issue, oversampling was done for the minority classes, and under-sampling was done for the majority class. Such approaches further help the Siamese Network deal with class imbalance inherently, learning from pairs of images and resulting in balanced precision and recall across classes. We experimented with the number of training epochs at 20, 40, and 60. Increasing the training epochs presented better performance in the network, but the results started stabilizing when the training epochs went above 40, indicating that all important features have been captured around that time.

## 8. Conclusion

In this paper, we came up with a way to detect MI using Siamese Network Model for detecting cardiovascular diseases from ECG Signals.

- In this proposed methodology one does not have to modify the ECG signals to keep the manual disturbance and noise to a minimum as possible and the results as authentic as possible
- The time complexity for training with the Siamese Network Model is mainly O(n^2), as it involves making pairwise comparisons. Actual training on the dataset of 928 ECG images took approximately 12 hours on Google Colab with an NVIDIA T4 GPU, while inference for each image pair was under 100 milliseconds, ensuring real-time applicability.
- Our authentic machine-learning architecture ensures the novelty of the solution.
- Since we tried several machine learning algorithms but when we used CNN along with the Siamese network it gave us extraordinary results due to which we found out that this new architecture works best to detect MI using ACG signals.
- According to the result we got from training these models and testing them on several datasets, we can ensure high accuracy and positive prediction of MI.

For future work, we aim to modify our system for real-time detection.

## Abbreviations

SNN: Siamese Neural Networks
MI: Myocardial Infarction
ECG: Electrocardiogram
CVD: Cardiovascular Diseases
MRI: Magnetic Resonance Imaging
DCNN: Deep Convolutional Neural Network
CNN: Convolutional Neural Network
CAM: Class Activation Mapping
EMD: Empirical Mode Decomposition
LMT: Logistic Model Trees
